# Glutamine ameliorates *Bungarus multicinctus* venom-induced lung and heart injury through HSP70: NF-κB p65 and P53/PUMA signaling pathways involved

**DOI:** 10.1590/1678-9199-JVATITD-2022-0080

**Published:** 2023-07-10

**Authors:** Yalan Li, Zhezhe Guan, Shaocong Hu, Zhi Huang, Dongling He, Xiaoyang Cheng, Tianlin Song, Caifeng Mo, Manqi Xiao, Yue Huang, Yuanmei Wei, Yi Zhou, Xuerong Zhang, Ming Liao

**Affiliations:** 1 Guangxi Medical University, Nanning, PR China.; 2 The First Affiliated Hospital of Guangxi University of Chinese Medicine, Nanning, PR China.; 3 Dinghu People’s Hospital, Zhaoqing, PR China.; 4 Tongji University Cancer Center, Shanghai Tenth People’s Hospital, Tongji University School of Medicine, Shanghai, PR China.

**Keywords:** Glutamine, *Bungarus multicinctus* bite, HSP70, NF-κB p65, P53/PUMA

## Abstract

**Background:**

*Bungarus multicinctus* is one of the most dangerous venomous snakes prone to cardiopulmonary damage with extremely high mortality. In our previous work, we found that glutamine (Gln) and glutamine synthetase (GS) in pig serum were significantly reduced after *Bungarus multicinctus* bite. In the present study, to explore whether there is a link between the pathogenesis of cardiopulmonary injury and Gln metabolic changes induced by *Bungarus multicinctus* venom. We investigated the effect of Gln supplementation on the lung and heart function after snakebite.

**Methods:**

We supplemented different concentrations of Gln to mice that were envenomated by *Bungarus multicinctus* to observe the biological behavior, survival rate, hematological and pathological changes. Gln was supplemented immediately or one hour after the venom injection, and then changes in Gln metabolism were analyzed. Subsequently, to further explore the protective mechanism of glutamine on tissue damage, we measured the expression of heat-shock protein70 (HSP70), NF-κB P65, P53/PUMA by western blotting and real-time polymerase in the lung and heart.

**Results:**

Gln supplementation delayed the envenoming symptoms, reduced mortality, and alleviated the histopathological changes in the heart and lung of mice bitten by *Bungarus multicinctus*. Additionally, Gln increased the activity of glutamine synthetase (GS), glutamate dehydrogenase (GDH) and glutaminase (GLS) in serum. It also balanced the transporter SLC7A11 expression in heart and lung tissues. *Bungarus multicinctus* venom induced the NF-κB nuclear translocation in the lung, while the HO-1 expression was suppressed. At the same time, venom activated the P53/PUMA signaling pathway and the BAX expression in the heart. Gln treatment reversed the above phenomenon and increased HSP70 expression.

**Conclusion:**

Gln alleviated the glutamine metabolism disorder and cardiopulmonary damage caused by *Bungarus multicinctus* venom. It may protect lungs and heart against venom by promoting the expression of HSP70, inhibiting the activation of NF-κB and P53/PUMA, thereby delaying the process of snake venom and reducing mortality. The present results indicate that Gln could be a potential treatment for *Bungarus multicinctus* bite.

## Background

Snakebite is a serious public health issue in many regions of the planet [1]. It is reported that there are more than 5 million people suffer from snake bites annually around the world, of whom 1.8 to 2.7 million people develop clinical illness and 81,000 to 138,000 die from complications [2-5]. Bites by *Bungarus multicinctus* are a health problem especially in tropical regions. This animal is one of the top ten venomous snakes in China [6, 7]. Although the frequency of its bites is not very high, the mortality rate is higher than that of any other venomous snake in China [8, 9]. Local symptoms and signs of victims bitten by *Bungarus multicinctus* usually do not include serious swelling or pain. Consequently, victims are often misidentified as having been bitten by a non-venomous snake which results in mistimed treatment [10].

*Bungarus multicinctus* venom mainly contains neurotoxins, hematotoxins and cardiotoxins. Neurotoxins block nerve conduction and cause hypoxia indirectly leading to heart and lung damage, while hematotoxins and cardiotoxins cause direct damage to cardiopulmonary tissue [11, 12]. Within 1h to 6h, patients may have symptoms of systemic envenoming, respiratory failure, pulmonary interstitial edema, heart failure, cardiopulmonary function damage, and even multiple organ failure (MOF), which seriously endanger their lives [13]. Although antivenom has a high ability to neutralize free snake venom, it cannot neutralize toxins that are bound to cardiopulmonary tissue cells. Further progression of cardiopulmonary injury is prone to MOF [8, 14]. Therefore, it is necessary to investigate the treatment of the cardiopulmonary injury caused by *Bungarus multicinctus* venom in order to inhibit the development of snakebite syndrome.

In our previous work, the combination of untargeted and targeted metabolomics found that the metabolite glutamine (Gln) and glutamine synthetase (GS) concentrations were significantly lower in the serum of pigs bitten by *Bungarus multicinctus*. Moreover, the histological examination of the lung and heart showed the most significant toxic changes [15]. However, the inner link between Gln metabolism changes and tissue damage after venom was not revealed. 

Gln is the most abundant and versatile amino acid in the body and plays a critical role in nitrogen exchange among organs, intermediary metabolism, immunity, and pH homeostasis [16]. By definition, Gln is a non-essential amino acid but has been noted to be “conditionally essential” during pathological stress such as pathogen infection and starvation. In tissue and blood, Gln concentrations are dependent on glutamine synthetase (GS), phosphate-dependent glutaminase (GLS) and glutamate dehydrogenase (GDH) activities [17]. The dynamic change of Gln transporters can directly affect intracellular Gln content. When the Gln consumption is higher than the output, the application of dipeptide alanyl-glutamine, as an effective Gln supplement, increased plasma Gln in patients. 

Numerous experimental and clinical trials have demonstrated that Gln protects lung and heart, including protecting ischemia-reperfusion induced acute lung injury in isolated rat lungs and modulating endothelial progenitor cell and lung injury in septic mice [18, 19]. Gln has been shown to increase load tolerance in patients with ischemic heart disease. Gln anaplerosis might not only contribute to cardiac mitochondrial energy generation, but also enhance antioxidant synthesis, further contributing to cardiac protection. Gln treatment also reduces myocardial cell apoptosis in sepsis rat [20, 21]. 

Many studies have revealed that inflammatory mediators, oxidative stress, and apoptosis are the main factors of lung and heart injury. NF-κB p65 and P53 are key transcription factors in the regulation of inflammatory response and apoptosis, respectively [22]. NF-κB p65 upregulates the pro-inflammatory molecules, such as tumor necrosis factor alpha (TNF-α), interleukin (IL-1β) and toll-like receptor (TLR-4) [23]. P53 upregulates the expression of the pro-apoptotic genes, including Bax, Fas and Fas ligand (FasL) which activate apoptosis [24]. The HSP70 high levels attenuated the production of inflammatory cytokines by inactivation of NF-κB p65 [25]. On the one hand, both *in vitro* and *in vivo* research have reported that Gln-induced HSP70 triggered the release of anti-inflammatory cytokines and reduced inflammatory factors via the NF-κB p65 pathway, thereby attenuating lung injury after sepsis. On the other hand, Gln can also protect cardiomyocytes by regulating the P53 signaling pathway to inhibit cell apoptosis [26, 27].

The lung and heart are among the most vulnerable organs to *Bungarus multicinctus* envenomation. Unfortunately, there are few reports on the relationship between inflammation, apoptosis and metabolic alteration in lung and heart dysfunction caused by *Bungarus multicinctus* bites. Thus, following our previous studies, we tried to investigate whether supplementation of Gln can protect mice experimentally envenomated by *Bungarus multicinctus* and reveal the role of Gln in *Bungarus multicinctus* bite induced inflammatory response and apoptotic change in mouse lungs and heart.

## Methods

### Materials

The main reagents used in this study were the following: Alany Glutamine Injection was manufactured by Fresenius Kabi S.A. The venom of *Bungarus multicinctus* was supplied by the Snake Venom Institute, Guangxi Medical University. The IL-10, TNF-a, SLC7A11 and SLC1A5 levels were quantified using an enzyme-linked immunosorbent assay (ELISA) kit (CUSABIO, Wuhan, China). The Gln, GS, GLS, GDH, SOD and MDA levels were quantified using a kit (Nanjing Jiancheng Bio-Technology Co., Ltd.).

### Animals

A total of 140 healthy male mice, weighing 18 ?? 2g and aged between 5 and 6 weeks were obtained from the experimental Animal Center of Guangxi Medical University. They were fed in separate cages in the specific pathogen-free animal room with a standard 12h-light and 12h-dark cycle and *ad libitum* access to food and water. The used animal experimental protocol was under the approval of Ethics Committee of Guangxi Medical University (ethical review number NO:202102002).

Mice were injected with snake venom intramuscularly in the thigh, as to simulate a typical snake bite, and intraperitoneally injected with different concentrations of Gln (n = 20 for each group):

- Control group: mice were injected with PBS.
- Venom group: mice were intramuscularly injected with snake venom and intraperitoneally injected with PBS.
- Venom+LGln: mice were intramuscularly injected with snake venom and intraperitoneally injected with Gln (0.1 g/kg).
- Venom+MGln: mice were intramuscularly injected with snake venom and intraperitoneally injected with Gln (0.3 g/kg).
- Venom+HGln: mice were intramuscularly injected with snake venom and intraperitoneally injected with Gln (0.5 g/kg).

Ten mice in each group were observed at regular intervals for occurrence of mortality and biological behavior over the subsequent 24 hours. For further experiments, mice were divided into four groups of 10 mice each, immediately after venom injection, or at 1h after envenoming, a dose of 0.5 g/kg of Gln intraperitoneal injection:

- Control group: mice were injected with PBS.
- Venom group: mice were intramuscularly injected with snake venom and intraperitoneally injected with PBS.
- Venom+Gln 0h group: mice were intramuscularly injected with snake venom and Immediate intraperitoneal injection of Gln.
- Venom+Gln 1h group: mice were intramuscularly injected with snake venom and intraperitoneal injection of Gln after 1h.

All animals were anaesthetized with isoflurane inhalation and killed by high concentration of carbon dioxide at 4 h after *Bungarus multicinctus* venom administration.

### Biological behavior and survival rate

Initial experiments were designed to determine whether Gln can relieve the symptoms of intoxication. With this purpose, the biological behavior of mice, such as movement status, mental status and respiratory performance were observed. Mice from all experimental groups monitored every 4h during 24h after venom. All mice had unrestricted access to food and water. The results are expressed as percentage (%) of survival. 

### Hematological indexes

The blood was collected from the eyeball of mice 3h after the injection of snake venom. The blood in the EDTA anticoagulant tube was inverted 5-8 times to ensure even mixing, and then the blood routine and other indicators were detected by a BC-5390 automatic blood routine analyzer (Shenzhen, China). The blood in the ordinary serum tube was naturally coagulated at room temperature for 1h, and then centrifuged at 3000 rpm for 15 minutes at 4℃. After the serum was separated, the blood biochemical level was detected by the BS-2000 automatic biochemical analyzer (Shenzhen, China).

### Hematoxylin and eosin histochemical staining

Lung and heart tissues were removed. Then post-fixed for 24h by 10% formaldehyde and processed for paraffin embedding. After routine processing, paraffin sections of each tissue were cut into 5mm thickness and stained with hematoxylin and eosin (H&E). Selected H&E stained slides were examined with a light microscope (Nikon Eclipse E600, Japan). Interstitial edema, inflammatory cell infiltration and myofibrillerlysis were examined histopathologically. According to Szapiel [28] and Tirilomis et al. [29] standards, the evaluation of lung and heart tissue includes four categories from 0 (normal) to 3 (severe).

### Enzyme-linked immunosorbent assays

The levels of IL-10 and TNF-α in the blood samples were measured using ELISA kits (Cusabio Biotech Co. Ltd. Wuhan, China.), according to the manufacturer's protocols. Briefly, the kits were used for determination after keeping at room temperature for at least 20 minutes. The samples were added into each well and incubated for 2 h. The biotin-antibody was then added and incubated for another 1 h at 37°C. Next, horseradish peroxidase-conjugate was immediately added into the well followed by incubation for 1 h. Subsequently, the chromogenic substrate and stop solution were incubated in the dark, respectively. The absorbance was finally measured with a microplate reader at wavelength of 450 nm (Thermo Fisher Scientific Inc., Waltham, MA, USA).

### TUNEL assay and immunohistochemistry for lung and heart tissue

Paraffin-embedded lung and heart tissues were processed for immunochemistry. Apoptotic cells were detected by TdT-mediated dUTP nick end labeling (TUNEL) method. The TUNEL assay was carried out according to the manufacturer’s instructions (Roche Applied Sciences, Shanghai, China). Then TUNEL-positive cells were counted in six random sections. Lung tissues and heart tissues were fixed in 4% formalin and embedded in paraffin as previously described. About 5-μm lung paraffin sections were dewaxed, hydrated, and then incubated with anti-HSP70 antibody (diluted 1: 200; Cell Signaling Technology, USA) at 4°C overnight. After biotin-labeled secondary antibody was added to the slides, slides were stained with 3,3’-diaminobenzidine (DAB) and counterstained with hematoxylin. The stained slides were observed by using a digital camera under microscope (Leica, DMLB2, Germany). Finally, immunoreactive cell percentage was visually scored as 0 (none), 1 (< 10%), 2 (10-50%), 3 (51-80%), or 4 (> 80%). Staining intensity was scored as 0 (none), 1 (weak), 2 (moderate), or 3 (strong). Combined scores for each specimen were calculated by multiplying percent immunoreactivity and staining intensity values (possible range: 0-12).

### The W/D, SOD and MDA levels in lungs

The left lobe was used for measurement of lung water content. Lung lobes were weighed before (wet weight) and after (dry weight) drying for 24 h in an 80°C oven. The water content of the lung was calculated as: lung water content = wet weight/dry weight.

Lung tissues were prepared as 10% tissue homogenates and centrifuged at 3000 rpm for 10 minutes at 4°C. The SOD activity was measured using the Water soluble tetrazole salt method. The MDA content was measured by the thiobarbutiric acid colorimetric method. The kits were used according to the manufacturer’s instructions (Nanjing Jiancheng Bioengineering Institute, Nanjing, China). The absorbance was measured at 450 and 532 nm, respectively, using an ultraviolet spectrophotometer.

### Gln metabolism analysis

*Glutamine content in serum, lungs and heart*

The blood samples were centrifuged at 6000 rpm for 10 minutes at room temperature, and the supernatants were subsequently collected and stored at -20°C. Lung and heart tissues were prepared as 10% tissue homogenates and centrifuged at 3000 rpm for 10 minutes at 4°C. The content of Gln was detected using commercially available kits (Nanjing Jiancheng Bioengineering Institute, Nanjing, China) according to the manufacturer’s recommended protocol.

*Quantification of GLS, GS and GDH activity in serum*

The quantification of GLS, GS and GDH activity were detected using commercially available kits (Nanjing Jiancheng Bioengineering Institute, Nanjing, China) according to the manufacturer’s recommended protocol.

### 2.9.3. Protein contents of SLC7A11 and SLC1A5 in lungs and heart

The levels of SLC7A11 and SLC1A5 in the tissues were measured using ELISA kits (J&L Biological, Shanghai, China).

### Reverse transcription-quantitative polymerase chain reaction (RT-qPCR)

In order to investigate the relative mRNA expression of HSP70, HO-1, NF-κB p65 and IκB-β in lung tissue, HSP70, P53, PUMA in heart tissue. Total RNA was extracted using TRIzol Reagent (Thermo Fisher Scientific, Inc. USA). The thermocycling conditions for PCR were as follows: Initial denaturation step at 95°C for 30 seconds; followed by 40 cycles of denaturation at 95°C for 5 seconds, annealing at 60°C for 34 seconds; and a dissociation stage at 95°C for 15 seconds, followed by 60°C for 1 minute and 95°C for 15 seconds. The primers used for amplification of the respective genes are listed in Table 1.

**Table 1.** List of primer sequences used in quantitative polymerase chain reaction.

Gene	Primer sequence (5'-3')	
	Forward	Reverse
β-actin	GTGCTATGTTGCTCTAGACTTCG	ATGCCACAGGATTCCATACC
HSP70	CGCTCGAGTCCTATGCCTTCA	GGCACTTGTCCAGCACCTTC
HO-1	TCCTTGTACCATATCTACACGG	GAGACGCTTTACATAGTGCTGT
NF-κBP65	TCGAGTCTCCATGCAGCTACGG	CGGTGGCGATCATCTGTGTCTG
IκB-β	ACCTCACTCAGAGCCAGGAC	GCCTCCAGTCTTCATCACGC
P53	ACCTTATGAGCCACCCGAGG	AAGGATAGGTCGGCGGTTCA
PUMA	GCGTGTGGAGGAGGAGGAGTG	CCAGGGTGAGGGTCGGTGTC

### Western Blot Assay

In order to determine the activation state of the NF-κB and P53/PUMA signaling pathways, the lung and heart tissues were washed in ice-cold saline, they were homogenized in 4℃ RIPA lysis buffer (with 1 mM PMSF). The nuclear and cytoplasmic proteins were extracted from the tissues. Next, they were centrifuged at 3000 g at 4℃ for 15 minutes and the supernatants were collected for assay. After the protein concentration was determined, 80 mg protein sample was loaded per lane and separated on SDS-PAGE. The target protein was then electrophoretically transferred to nitrocellulose membranes, which were blocked in TBST (5% nonfat milk, 10 mM Tris, 150 mM NaCl, 0.05% Tween-20) for 1 h. Next, they were blocked with first antibodies (Cell Signaling Technology, CA, USA) in 5% BSA wash buffer at 4℃ overnight, then treated with secondary antibody anti-rabbit IgG (1:10000) in TBST solution for 1 h. The blots were scanned by a two-color infrared imaging system (Odyssey; LI-COR Biosciences, Lincoln, NE, USA). Densitometry analysis was performed using Odyssey Software, version 3.0.29 (LI-COR Biosciences)

### Statistical analysis

All data are presented as mean ± standard error of the mean (SEM) from three independent experiments. Data were analyzed using one-way analysis of variance (ANOVA) followed by Tukey’s post hoc test in SPSS13.0 (Chicago, IL, USA). Value of p < 0.05 was considered to be significant between groups.

## Results

## Gln reduced mortality and protected lung and heart tissue in mice after *Bungarus multicinctus* bite

*Clinical manifestations and survival rate*

**Figure 1.**
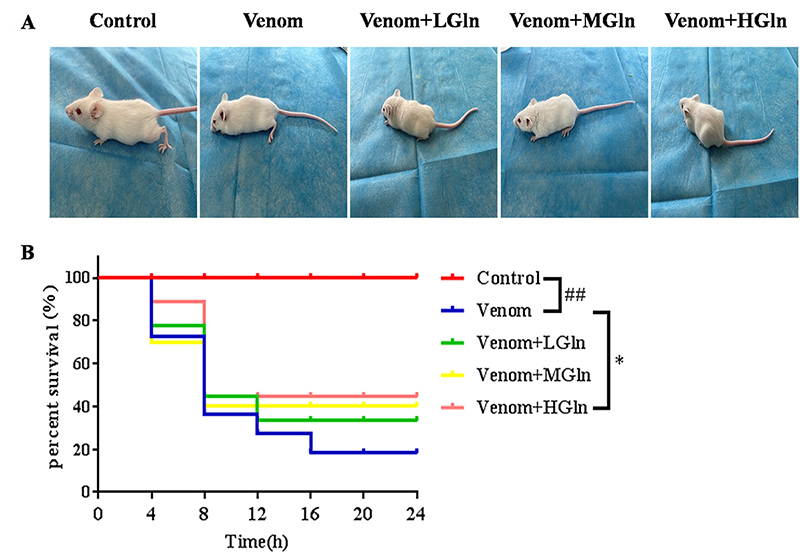
(A) Biological behavior changes three hours after injection with venom and Gln. (B) Survival rate of Gln-treated mice bitten by *Bungarus multicinctus*. ##p < 0.01 vs. control group; *p < 0.05 vs. venom group.

Compared with the control group (Figure 1A), the biological behavior results showed that 3 h after the injection of *Bungarus multicinctus* venom, the mice in the venom group clearly exhibited envenoming symptoms, such as ptosis, dyspnea, paralysis of the extremities. It is consistent with that of envenomation by *Bungarus multicinctus* in clinical practice. The Venom+LGln group also showed signs of envenoming with ptosis and quadriplegia. Mice in the Venom+MGln group exhibited a lesser degree of abdominal breathing, paralysis of the hind legs, and were able to crawl slowly. In contrast, the mice in the Venom+HGln group were in better mental condition, had less quadriplegia and were able to move forward with their hind legs. At the end of the observation period (24 h), 90% of venom-treated mice were dead. However, treatment with Gln, the mice mental state improved and mortality reduced (Figure 1B). Compared with venom group, Venom+HGln group developed abdominal breathing later and could crawl. These results indicate that Gln has an inhibitory effect on *Bungarus multicinctus* venom development.

*Hematological examination results*

Hematological changes are used to reflect the envenoming situation. Table 2 depicts the effect of treatment with Gln on the white blood cell (WBC), red blood cell (RBC), high-sensitivity C-reactive protein (CRP) and HCO_3_^-^. As compared to the control group, the level of WBC (p < 0.05) in the venom group decreased. The level of CRP (p < 0.05) and HCO_3_^-^ (p < 0.01) present the opposite trend to WBC. Nevertheless, the WBC were significantly increased in the Venom+MGln and Venom+HGln group (p < 0.05), the CRP and HCO_3_^-^ decreased significantly in the Venom+ HGln group (p < 0.05) when compared with Venom group. 

**Table 2.** Different concentrations of Gln affect the hematological index of mice three hours after snake venom injection.

Groups	WBC	RBC	CRP	HCO_3_^-^
Control	4.85 ?? 0.68	8.32 ?? 0.74	2.46 ?? 0.78	20.59 ?? 1.43
Venom	3.33 ?? 0.87#	7.37 ?? 0.38	3.67 ?? 0.44#	23.50 ?? 1.24##
Venom+LGln	4.25 ?? 0.68	8.29 ?? 0.88	3.05 ?? 1.23	22.09 ?? 1.65
Venom+MGln	4.40 ?? 0.92*	8.24 ?? 1.45	2.60 ?? 0.96	21.90 ?? 1.05
Venom+HGln	4.51 ?? 0.76*	8.20 ?? 0.65	2.50 ?? 0.73*	21.49 ?? 2.22*

Data are presented as mean ± SEM. #p < 0.05, ##p < 0.01 vs. control group; *p < 0.05, **p < 0.01 vs. venom group.

*Hematoxylin and eosin histochemical staining results*

**Figure 2.**
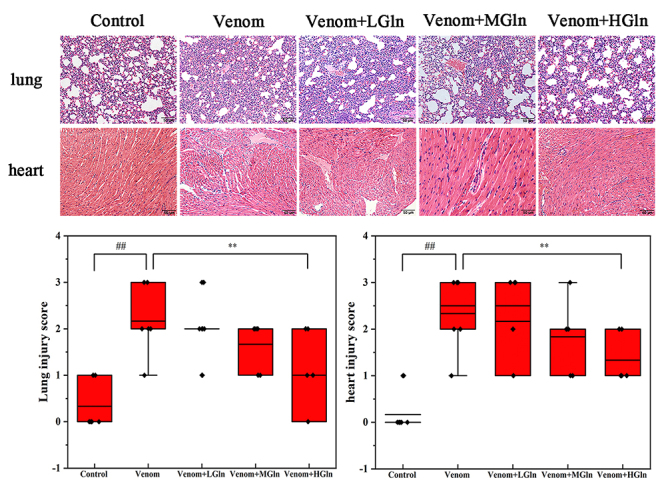
Calculated inflammation score in lung and heart tissues. Gln attenuates heart and lung injury after venom. Tissue sections were stained with hematoxylin and eosin. Original magnification: lung 20×; heart 20×. ##p < 0.01 vs. control group; **p < 0.01 vs. venom group.

As indicated in Figure 2, lung and heart sections in the control group showed normal architecture and no cellular influx. *Bungarus multicinctus* venom-induced lung resulted in destruction of alveolar structures, neutrophil infiltration, hyperemia, intra-alveolar hemorrhage, pulmonary edema and thickening of the septal space. The histopathological changes of myocardial tissue, including irregular cell arrangement, condensed cell nuclei and myocardial edema were seen in the venom group. Compared to the venom group, lung and heart edema and infiltration of inflammatory cells were diminished in the Gln treated group. Lung and heart tissue injury score results showed that the core of venom group was significantly higher than that of control group, and only the score of Venom+HGln group was significantly lower than that of the venom group (p < 0.01) (Figure 2). The 0.5 g/kg Gln has better protective effect on heart and lung tissue than 0.1 g/kg and 0.3 g/kg Gln. Therefore, we chose 0.5 g/kg Gln for follow-up research.

### Changes in Gln metabolism

*Glutamine levels in serum, lungs and heart*

**Figure 3.**
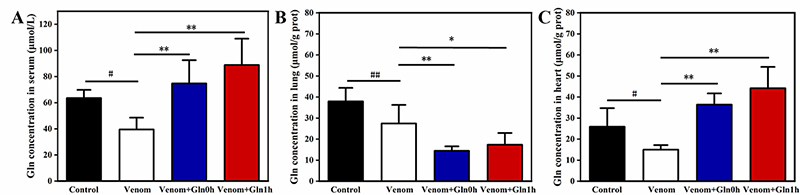
(A) Glutamine levels in serum. (B) Glutamine levels in lung. (C) Glutamine levels in the heart. #p < 0.05, ##p < 0.01 vs. control group; *p < 0.05, **p < 0.01 vs. venom group.

The results are shown in Figure 3, the changes of Gln content in serum and heart tissue were consistent. In serum and heart (Figure 3A and C)), snake venom significantly reduced the level of Gln compared to the control group (p < 0.05). After Gln supplementation, Gln levels were significantly increased (p < 0.01). In lung tissue (Figure 3B), Gln content was also significantly lower in the venom group (p < 0.01). However, Gln content was further reduced after Gln supplementation compared to the venom group (p < 0.05 or p < 0.01).

*The GS, GLS and GDH activity in serum*

**Figure 4.**
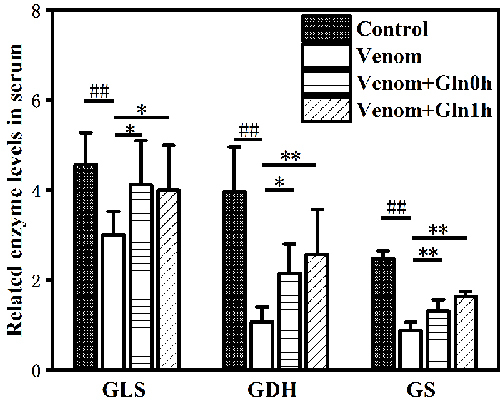
Effect of Gln on venom-induced activation of GLS, GDH and GS in serum. ##p < 0.01 vs. control group; *p < 0.05, **p < 0.01 vs. venom group.

Several enzymes are involved in Gln metabolism, the three main intracellular enzymes are Gln synthetase (GS), phosphate-dependent glutaminase (GLS) and glutamate dehydrogenase (GDH). As shown in Figure 4, GLS, GDH and GS enzyme activity was significantly decreased in serum by venom injection after 3 hours (p < 0.01). The enzyme activity of both the Venom+Gln 0h group and the Venom+Gln 1h group increased significantly after Gln supplementation (p < 0.05), maintaining the activity of serum GS, GLS and GDH close to basal levels observed in the control group.

*The SLC7A11 and SLC1A5 content in lung*

**Figure 5.**
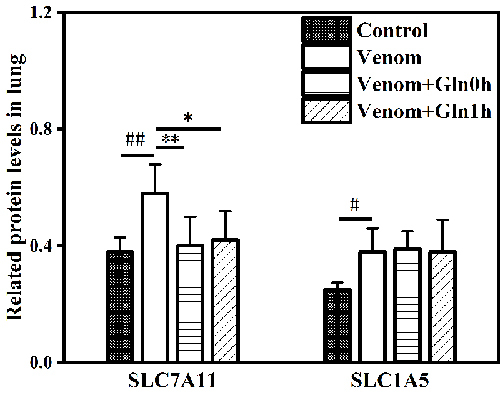
Effect of Gln on venom-induced content of SLC7A11 and SLC1A5 in lungs. #p <0.05, ##p < 0.01 vs. control group; *p < 0.05, **p < 0.01 vs. venom group.

In the lung tissue, venom promoted a significant increase in SLC7A11 and SLC1A5 expression compared with the control group (p ＜ 0.05 or p ＜ 0.01). However, the expression of SLC7A11 was attenuated both in the venom+Gln 0h and venom+Gln 1h groups compared with the venom group (p ＜ 0.05), while there was no significant change in SLC1A5 (p ＞ 0.05) (Figure 5). 

*The SLC7A11 and SLC1A5 content in the heart*

**Figure 6.**
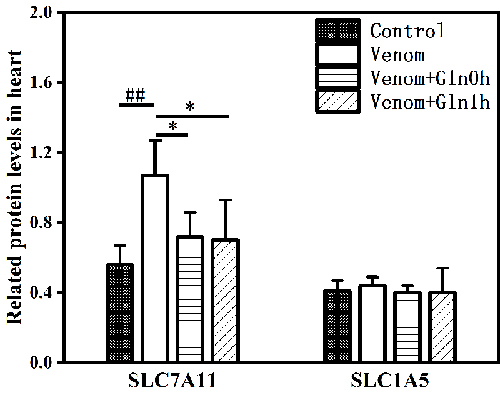
Effect of Gln on venom-induced content of SLC7A11 andSLC1A5 in the heart. ##p < 0.01 vs. control group; *p < 0.05 vs. venom group.

Venom similarly promoted the expression of SLC7A11 in heart tissue (p < 0.01). The SLC7A11 expression was diminished in the venom+Gln 0h and venom+Gln 1h groups compared with the venom group (p < 0.05) (Figure 6). However, SLC1A5 content did not show significant differences between the groups (p > 0.05).

### Gln treatment upregulated HSP70 expression, increased antioxidant levels, reduced inflammatory and apoptosis response in mice after *Bungarus multicinctus* venom

*Hematological examination results*

As depicted in Table 3, after the injection of *Bungarus multicinctus* venom, compared with the control group, the WBC in the venom group was significantly decreased (p < 0.05), while the CRP (p < 0.05) and HCO_3_^-^ (p < 0.01) concentrations were increased. When compared with the venom group, the WBC was increased, and HCO_3_^-^ was decreased in the Venom+Gln 0h group (p < 0.05), the CRP (p < 0.05) and HCO_3_^-^ (p < 0.01) were decreased significantly in the Venom+Gln 1h group. However, no significant difference was observed among the groups in the RBC. Our results showed that Gln administration attenuated infection, inflammation, myocardial function damage and respiratory function decrease may occur after *Bungarus multicinctus* bite.

**Table 3.** Gln supplementation at different times affects the hematological index in *Bungarus multicinctus* bitten mice.

Groups	WBC	RBC	CRP	HCO_3_^-^
Control	3.05 ?? 0.62	7.37 ?? 0.33	2.74 ?? 0.98	20.02 + 1.10
Venom	2.00 ?? 0.68#	5.89 ?? 1.20	3.80 ?? 0.50#	23.59 + 1.11##
Venom+Gln 0h	2.90 ?? 0.46*	7.23 ?? 0.40	2.84 ?? 0.86	21.52 + 1.92*
Venom+Gln 1h	2.30 ?? 0.36	6.82 ?? 0.56	2.60 ?? 0.62*	20.81 + 1.57**

Data are presented as mean ± SEM; #p < 0.05, ##p < 0.01 vs. control group; *p < 0.05, **p < 0.01 vs. venom group.

*Hematoxylin and eosin histochemical staining results*

**Figure 7.**
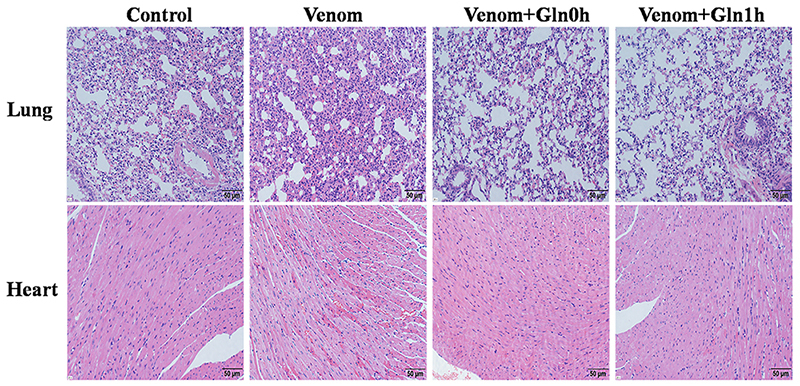
Effect of Gln on lung and heart after venom injury. Tissue sections were stained with hematoxylin and eosin. Original magnification: lung 20×; heart 20×.

The morphological changes of mice lung and heart tissues after different treatments were detected by light microscopy. As shown in Figure 7, compared to the control group, severe alveolar collapse, interstitial edema, and alveolar and mesenchymal hemorrhage were presented in the venom group. The histopathological changes of heart tissue, including irregular cell arrangement and condensed cell nuclei were seen in the venom group. These changes can be mitigated by supplementing Gln immediately or after envenoming symptoms.

*TUNEL and immunohistochemistry analysis*

**Figure 8.**
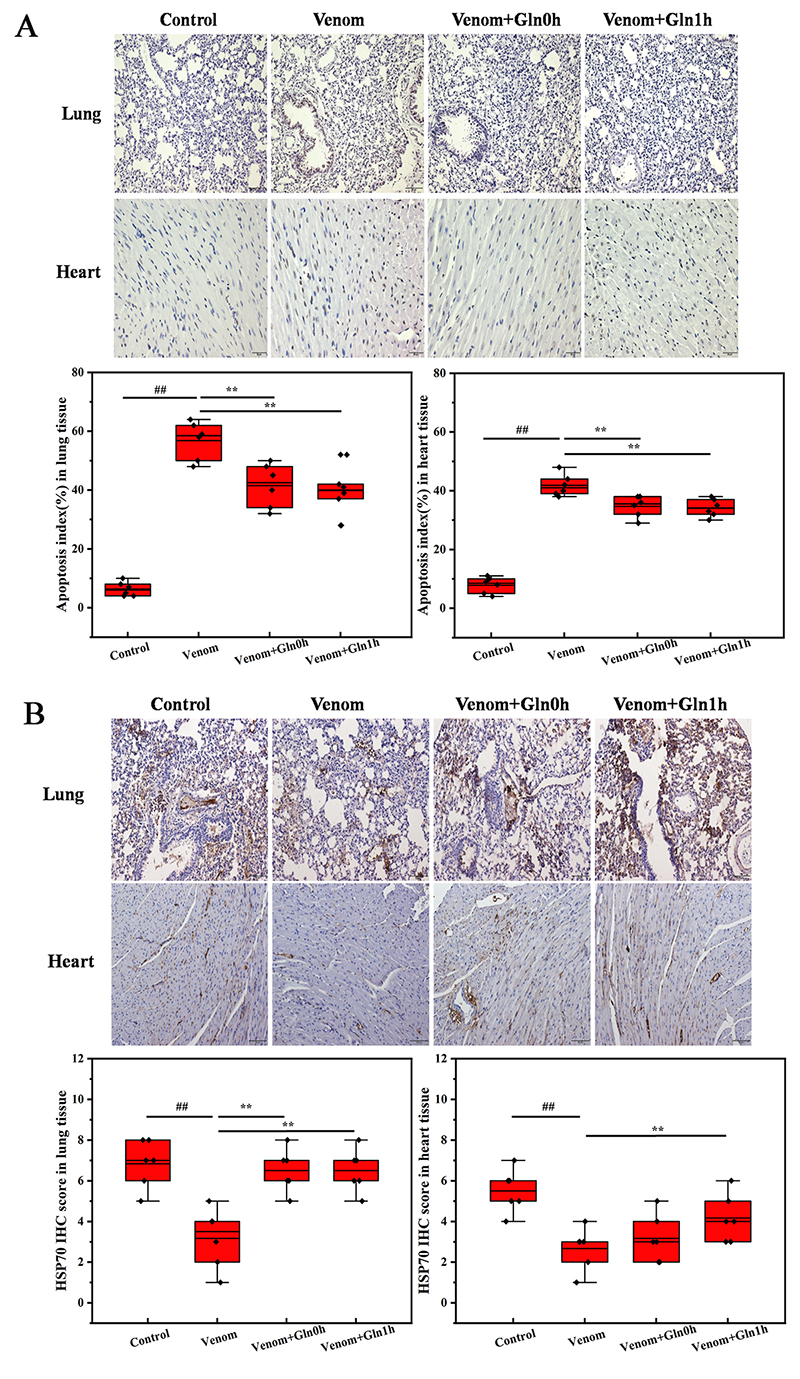
(A) Apoptosis changes of each group. (B) The expression of HSP70 changes of each group.

Cell apoptosis plays an important role in lung and heart injury. Compared to the control group, lung and heart cell apoptosis was significantly increased after venom injection, which indicated that venom-induced cell apoptosis may be an important factor leading to heart and lung dysfunction. As shown in Figure 8A, Gln treatment could significantly attenuate lung and heart apoptosis in Venom+Gln 0h and Venom+Gln 1h groups.

The HSP70 protein expression was measured by using the immunohistochemical technique, and results showed that HSP70 protein expression was significantly suppressed after venom treatment (Figure 8B). In the Venom+Gln 0h and Venom+Gln 1h group, the expression of HSP70 increased in the lung and heart.

*The W/D, SOD activity and MDA concentration in lung tissue*

**Figure 9.**
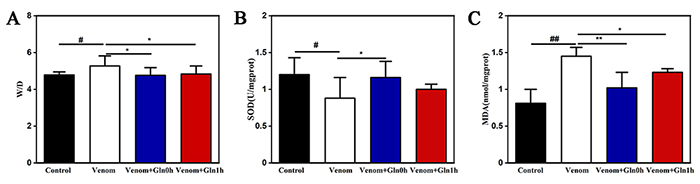
(A) Level of W/D in lung tissue. (B) Level of SOD activity in lung tissue. (C) Level of MDA concentration in lung tissue. #p < 0.05, ##p < 0.01 vs. control group; *p < 0.05, **p < 0.01 vs. venom group.

As shown in Figure 9A, levels of water content in lung tissues increased significantly in the venom group compared with the control group (p < 0.05). Both Venom+Gln 0h group and Venom+Gln 1h group after Gln supplementation significantly reduced water content (p < 0.05).

The lung tissue total SOD activity in the venom group was significantly lower (p < 0.05), and MDA concentration was significantly higher than those in the control group (p < 0.01). However, supplement with Gln significantly increased (p < 0.05) the total SOD activity and decreased (p < 0.01) the MDA concentration in Venom+Gln 0h group compared to the venom group. These results suggested that supplementation reversed pulmonary edema and oxidative damage (Figure 9A-B).

*Serum IL-10 and TNF-α levels*

**Figure 10.**
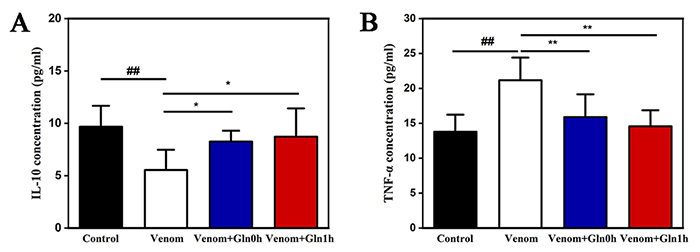
(A) Effect of Gln on venom-induced activation of IL-10. (B) Effect of Gln on venom-induced activation of TNF-a. ##p < 0.01 vs. control group; *p < 0.05, **p < 0.01 vs. venom group.

Compared with the control group, IL-10 activities were decreased in serum in the venom group (p < 0.01), which were reversed by Gln treatment in Venom+Gln 0h and Venom+Gln 1h groups (p < 0.05, Figure 10A). As shown in Figure 10B, serum levels TNF-α in the venom group were significantly higher than those in the control group (p < 0.01). The administration of Gln immediately or after 1 h significantly decreased serum TNF-α levels compared to the control group (p < 0.01). These results suggest that supplementation with Gln relieved inflammatory response imbalance.

### Gln increased HSP70 expression, regulated NF-κB p65 and P53/PUMA signaling pathways to protect lung and heart tissues

*HSP70 and NF-κB signaling pathway mRNA expression in lungs*

**Figure 11.**
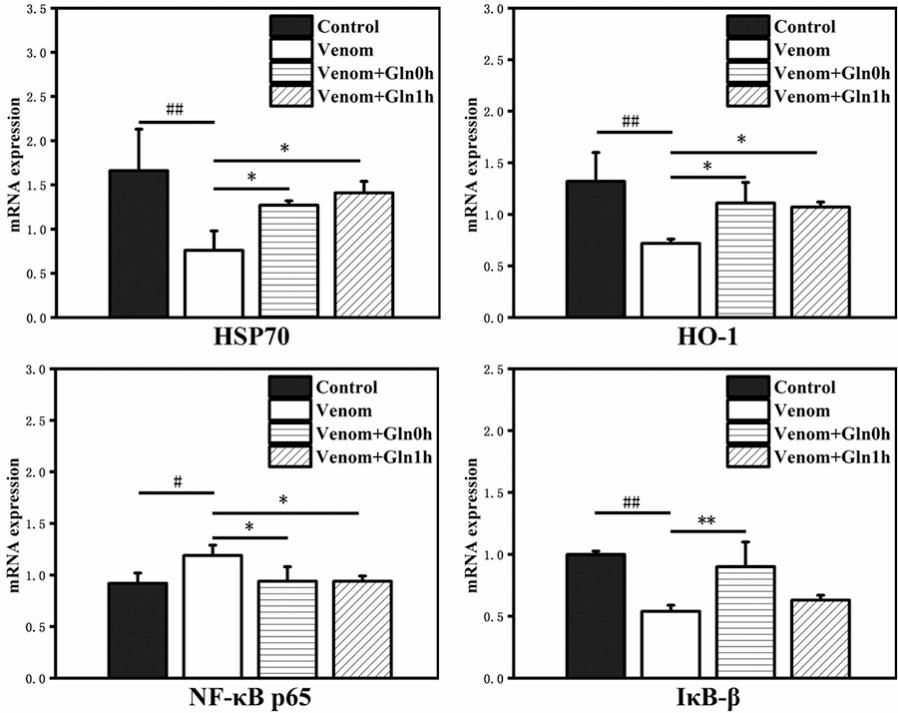
Effect of Gln on venom-induced mRNA expression of HSP70, HO-1, NF-κB p65, IκB-β in the lung. #p < 0.05, ##p < 0.01 vs. control group; *p < 0.05, **p < 0.01 vs. venom group.

NF-κB p65 signaling pathway is key regulators in inflammation. In the lung tissue, as compared to the control group, the increased mRNA expression of NF-κB p65 in venom group (p < 0.01). The mRNA expression of HSP70, HO-1, IκB-β present the opposite trend to NF-κB p65. By treatment with Gln in the Venom+Gln 0h and Venom+Gln 1h groups, the mRNA level of HSP70 and HO-1 were significantly increased compared with the venom group (p < 0.01). The mRNA expression of IκB-β in the Venom+Gln 0h group was also significantly increased (p < 0.01), while IκB-β in the Venom+Gln 1h group was not significantly changed compared with the venom group (Figure 11). 

*HSP70 and NF-κB signaling pathway protein expression in lungs*

**Figure 12.**
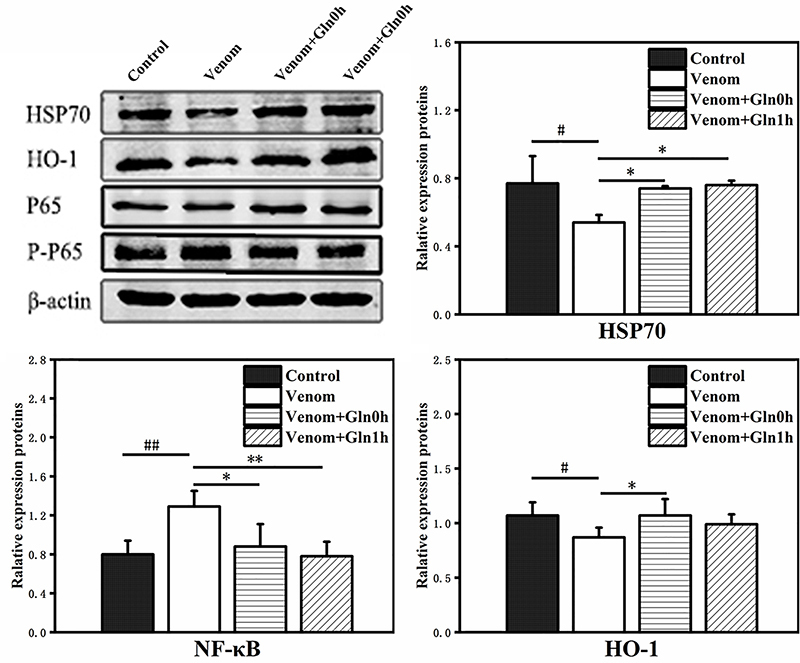
Effect of Gln on venom-induced mRNA expression of HSP70, HO-1, NF-κB p65, IκB-β in the lung. #p < 0.05, ##p < 0.01 vs. control group; *p < 0.05, **p < 0.01 vs. venom group.

The expression of HSP70 was consistent to the RT-qPCR result and our immunohistochemistry analysis data. In lung tissue, compared with the control group, the western blot result showed that HSP70 and HO-1 are significantly down regulated in venom group (p < 0.05), while the P-NF-κB p65/ NF-κB p65 was significantly increased (p < 0.01). Interestingly, Gln treatment increased the expression of HSP70 and HO-1 (p < 0.05), and decreased that of NF-κB p65 (p < 0.05) after snakebite. It suggested that HSP70 has important anti-inflammatory properties, providing venom tolerance by blocking the activation of the NF-κB pathway (Figure 12).

*HSP70 and P53/PUMA signaling pathway mRNA expression in the heart*

**Figure 13.**
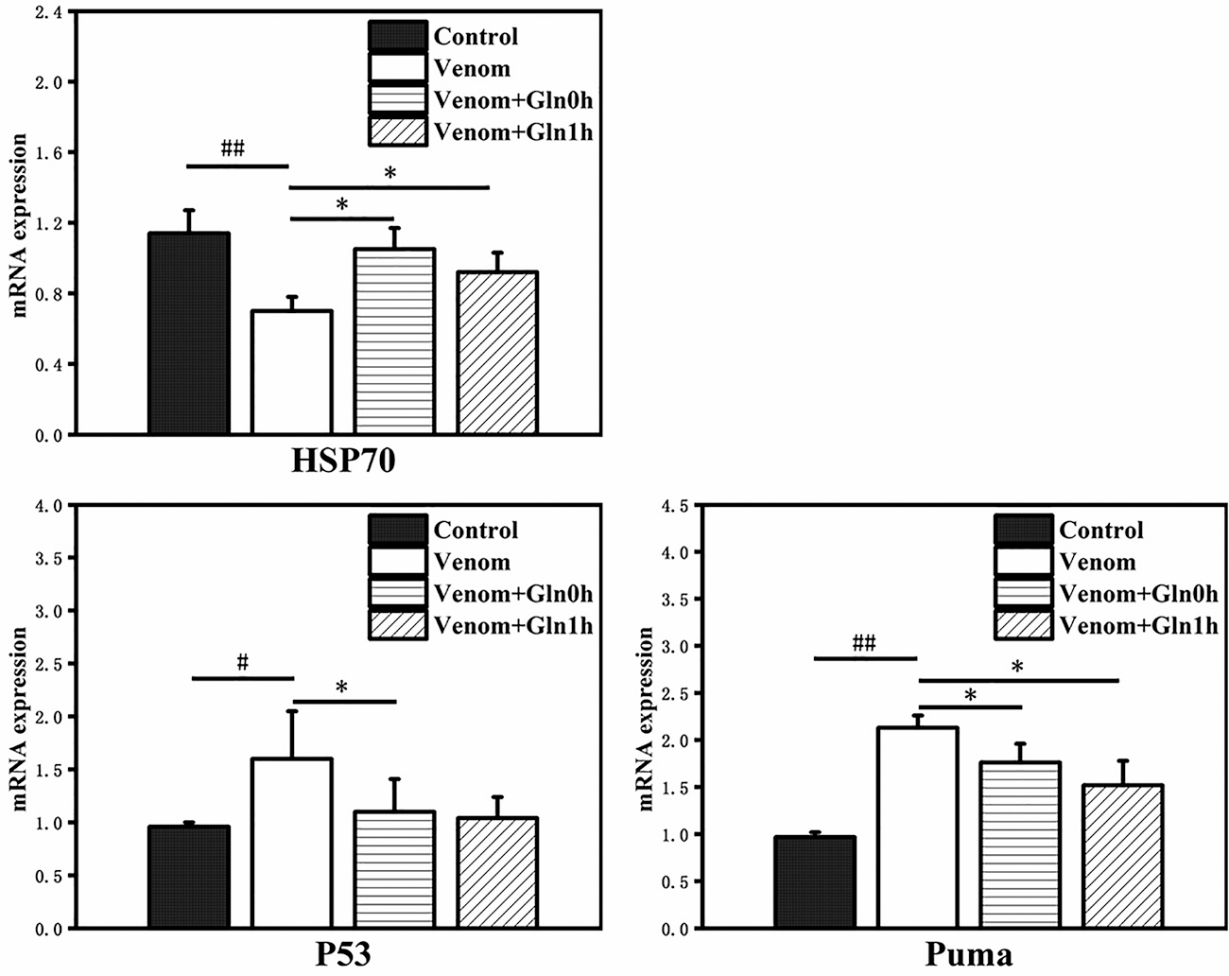
Effect of Gln on venom-induced mRNA expression of HSP70, P53, PUMA in the heart. #p < 0.05, ##p < 0.01 vs. control group; *p < 0.05 vs. venom group.

The P53 is key transcription factors in the regulation of apoptosis. The PUMA is a critical mediator of P53-dependent and -independent apoptosis in multiple tissues and cell types. In the heart tissue, HSP70 mRNA expression significantly decreased in the venom group in comparison with the control group (p < 0.01). The mRNA expression of P53 (p < 0.05) and Puma (p < 0.01) present the opposite trend to HSP70. By treatment with Gln, the HSP70 was significantly increased (p < 0.05), while P53 and PUMA were decreased compared with the venom group (p < 0.05). These data demonstrated that Gln prevented venom-induced heart tissue apoptosis through P53/PUMA (Figure 13). 

*HSP70 and P53/PUMA signaling pathway protein expression in the heart*

**Figure 14.**
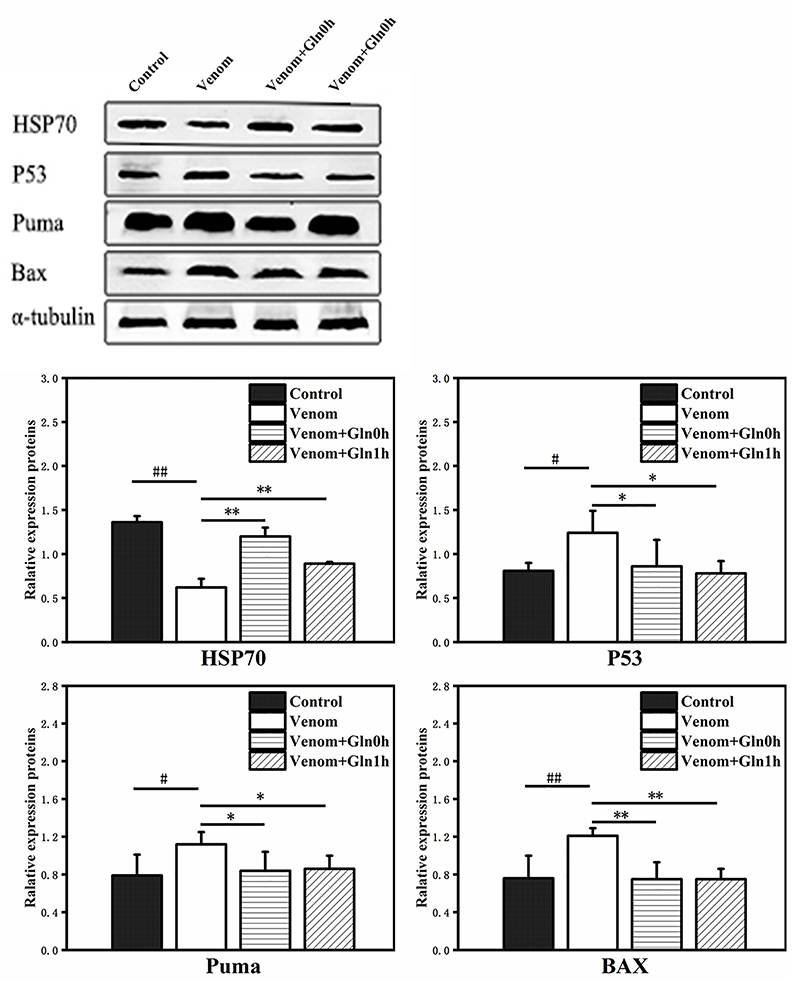
Effect of Gln on venom-induced protein expression of HSP70, P53, PUMA and BAX in the heart. #p < 0.05. ##p < 0.01 vs. control group; *p < 0.05, **p < 0.01 vs. venom group.

In the heart tissue, similarly, the protein expression of HSP70 was significantly decreased in the venom group compared with the control group (p < 0.01). Conversely, the protein expression of P53 (p < 0.05), PUMA (p < 0.05) and Bax (p < 0.01) were significantly increased. Supplementation with Gln significantly increased the expression levels of HSP70 (p < 0.01) compared with the venom group. However, the influence of Gln on P53, PUMA and Bax expression was contrary to the expression of HSP70. The expression level of P53 (p < 0.05), PUMA (p < 0.05) and Bax (p < 0.01) in the Venom+Gln 0h and Venom+Gln 1h groups were decreased significantly as compared to the venom group. These results suggest that supplementation with Gln rescued the venom-induced reduction of HSP70 and inhibited the activation of NF-κB and P53/PUMA signaling pathways in the lung and heart tissues (Figure 14).

## Discussion 

*Bungarus multicinctus* is a highly venomous species of the elapid snake. It regards as the most dangerous animal bitten disease in tropical and subtropical areas [30, 31]. Since the *Bungarus multicinctus* venom has the characteristics of a complex composition and the early symptoms of bites are not obvious. The further development of bites can easily result in severe damage to lung and heart function and cause multi-system organ failure (MOF) [6, 32]. At present, intravenous antivenom is the only specific treatment to counteract envenomation. However, antivenom cannot reverse lung and heart tissue that has been damaged by snake venom attacks [14]. Therefore, it is important to restore the cardiopulmonary function that was impaired by *Bungarus multicinctus* bite as soon as possible.

Studies have shown that in many lung and heart diseases, the interference of Gln can repair cardiopulmonary function [16, 17, 33]. In the lung, it had been reported that Gln improve the vascular function and ameliorate inflammation and damage of lung tissues [19, 34]. Gln treatment decreased IR-induced acute lung injury. The protective mechanism may be due to the inhibition of NF-κB activation and the attenuation of oxidative stress [35]. In the heart, many studies demonstrate that Gln supplementation protects against cardiometabolic disease, ischemia-reperfusion injury, sickle cell disease, cardiac injury by inimical stimuli, and may be beneficial in patients with heart failure [17]. In our previous works, we found that the metabolite Glutamine (Gln) was significantly lower in the serum of the *Bungarus multicinctus* bitten pigs. Moreover, the histological examination of the lung and heart showed the most significant toxic changes [15]. The composition of snake venom is complex and varies among different times or environments [7, 36]. Thus, we induced mice with the same venom as before and specifically supplemented Gln. The results found that different concentrations of Gln delayed the symptoms of venom and reduced mortality. We chose 500 mg/kg Gln to continue to explore the changes in Gln metabolism and specific protective mechanisms.

Glutamine cannot simply diffuse into cells across the plasma membrane; the transmembrane transfer of glutamine into cells needs mediation by transporters such as SLC7A11 and SLC1A5 [37]. Then, glutamine is converted into alpha-ketoglutarate (AKG) through two pathways, namely, the glutaminase (GLS) I and II pathway. AKG is an intermediate in the TCA cycle and functions as a source of energy for the cells. Reversely, AKG can be converted into glutamine by glutamate dehydrogenase (GDH) and glutamine synthetase (GS), or be converted into CO_2_ via the tricarboxylic acid (TCA) cycle and provide energy for the cells [38, 39]. Glutamine is also the precursor of glutathione (GSH) which may protect cells against oxidative injury [40, 41]. 

We investigated the levels of Gln, Gln metabolism-related enzymes and transporter proteins in serum and tissues. Of note, *Bungarus multicinctus* snake venom significantly reduced the level of Gln in the body, inhibited the activity of GS, GLS and GDH in serum, and promoted the expression of SLC7A11 in lung and heart tissues. The abnormal metabolism of Gln was reversed by Gln supplementation. As the mice showed symptoms of hypermetabolic systemic toxicity with irritability, convulsions and organ failure after the snake bite [30]. Therefore the organism's demand for Gln increased sharply and the content of Gln decreased significantly [17]. Gln deprivation affected the activity of Gln metabolizing enzymes, which further affected the TCA cycle and glutathione formation [42, 43]. Thus, cell energy synthesis was blocked, and at the same time suffered from ROS. Glutamine metabolism disorder can promote the transporter SLC7A11 expression in the lung and heart by positive feedback, so as to help cells rebuild redox homeostasis under stress [44]. 

Unexpectedly, the content of Gln in the lung tissue decreased rather than increased after Gln supplementation. We speculate that Gln supplementation protected Gln metabolizing enzymes in lung tissue. Meanwhile, the transporter protein SLC1A5 expressed highly to mediate Gln uptake. The TCA cycle and glutathione synthesis pathway were restored and Gln metabolism was enhanced. The intake of Gln was insufficient to balance lung tissue demand immediately. Overall, when the Gln content in the body is abundant after Gln supplementation, the Gln metabolism disorder is alleviated.

In order to further reveal the molecular mechanism of supplementing Gln to repair lung and heart injury caused by *Bungarus multicinctus* bite, we analyzed HSP70 protein, and key regulators of NF-κB p65 and P53/PUMA signaling pathways. HSPs are believed to serve as extracellular inflammatory messengers and intracellular cytoprotective molecules. Enhanced HSP expression is a key mechanism by which Gln confers protection in critical illness [22, 45-47]. It has been reported that lung tissue has an impaired ability to express HSP70 after sepsis, but Gln supplementation can correct this impairment [48]. Li W et al. [49] also showed that Gln mitigated smoke inhalation-induced lung inflammatory response, and further prevented the activity of NF-κB. More importantly, Gln enhanced the expression of HSF-1, HSP-70 and HO-1 in lung tissues. Further studies by Singleton et al. [35, 50] have shown that glutamine attenuates NF-κB activation by inhibiting Cullin-1 de-neddylation suggesting that the inhibition of NF-κB activation may be one of the mechanisms whereby glutamine may exert its protective effect. Collectively, these results indicate that Gln can promote the HSP70 expression and regulate NF-κB inflammation response to protect lung tissue. 

Our data shows that in the lung, whether glutamine was given immediately or after 1 hour in *Bungarus multicinctus* bite mice, western blot and immunohistochemistry both showed increased HSP70 expression. Similarly, HO-1, known as HSP-32, is also an inducible isoform in response to stress such as oxidative stress. We found that the HO-1 level also increased after Gln. All these indicate that, as a potent protective agent of organic injury, HSP70 and HO-1 was upregulated in response to cell stress and protected tissues and organs against *Bungarus multicinctus* venom injury. In addition, NF-κB p65 activation was completely inhibited by Gln, also the IκB-β degradation. Therefore, it is speculated that the protective mechanisms of glutamine may be that HSP70 inhibits NF-κB and regulates inflammation by stabilizing IκB degradation, thereby alleviating lung cell inflammation and preventing *Bungarus multicinctus* venom-induced acute lung injury progresses.

In heart disease research, early administration of glutamine protects cardiomyocytes from Post-Cardiac Arrest Acidosis [51]. In a CLP rat model, treatment with Gln reduced sepsis-induced cardiac myocyte injury [52]. The findings were that Gln decreased the cardiac myocyte apoptosis induced by sepsis through the promotion of HSP90 expression [26]. Glutamine enhances the heat shock protein 70 expression as a cardioprotective mechanism in left heart tissues in the presence of diabetes mellitus [53]. The expression levels of cell stress (p53) and apoptosis (caspase-3, bcl-xL) markers were significantly lower in cardiomyocytes treated with glutamine than those without glutamine [54]. Similarly, glutamine supplementation might not only contribute to cardiac mitochondrial energy generation, but also enhance antioxidant synthesis, further contributing to cardiac protection [20, 55]. The above studies pointed out that Gln can repair myocardial injury by mobilizing the apoptosis factor Bcl-2/Bax and affecting the expression of HSP70, P53 and PUMA. 

As shown in our heart tissue result, apoptosis, P53/PUMA and BAX activities were significantly decreased in venom mice following treatment with Gln, but HSP70 conversely. Therefore, we speculate that Gln may prevent or reduce cardiac myocyte apoptosis through promoting HSP70 expression or reversing the decreased of HSP70. Gln also regulates P53 and PUMA expression in the heart function repair. The PUMA may signal primarily to the mitochondria, where it acts indirectly on the Bcl-2 family members Bax by reducing the inhibition of apoptosis to protect heart tissue in *Bungarus multicinctus* bite. 

We have tested the hematological indexes. Glutamine alleviates the decrease in WBC and inhibits the increase in CRP and HCO_3_^-^ caused by *Bungarus multicinctus* venom. Besides, the venom-induced inflammation by production of pro-inflammatory molecules TNF-a and inhibiting the anti-inflammatory molecules IL-10. Gln supplements reversed this finding. It suggests that glutamine enhances the body's immunity, reduces infection and inflammation, and maintains the acid-base balance against the *Bungarus multicinctus* bite. 

## Conclusion 

In summary, the results demonstrated that supplementing Gln can redress Gln metabolic disorder, alleviate lung and heart edema, reduce the inflammatory mediator production and inhibit cell apoptosis after *Bungarus multicinctus* bite, to protect the lungs and heart. The Gln promoted the HSP70 expression, and inhibited NF-κB activation in the lung and P53/PUMA activation in the heart could be a potential mechanism involved in its protection. Therefore, the administration of Gln may be a useful prophylactic or adjusted drug therapy for *Bungarus multicinctus* venom-induced lung and heart injury, and further clinical were warranted.
